# Time-on-task effects in children with and without ADHD: depletion of executive resources or depletion of motivation?

**DOI:** 10.1007/s00787-017-1006-y

**Published:** 2017-05-23

**Authors:** Tycho J. Dekkers, Joost A. Agelink van Rentergem, Alette Koole, Wery P. M. van den Wildenberg, Arne Popma, Anika Bexkens, Reino Stoffelsen, Anouk Diekmann, Hilde M. Huizenga

**Affiliations:** 1 Department of Psychology, University of Amsterdam, Nieuwe Achtergracht 129B, 1018 WS Amsterdam, The Netherlands; 2 Department of Forensic Psychiatry and Complex Behavioral Disorders, Academic Center for Child and Adolescent Psychiatry, De Bascule, Rijksstraatweg 145, 1115 AP Duivendrecht, The Netherlands; 3 Amsterdam Brain and Cognition Center, University of Amsterdam, Amsterdam, The Netherlands; 4 Department of Child and Adolescent Psychiatry, VU University Medical Center Amsterdam, Amsterdam, The Netherlands; 5 Faculty of Law, Institute of Criminal Law and Criminology, Leiden University, Leiden, The Netherlands; 6 Department of Developmental and Educational Psychology, Leiden University, Wassenaarseweg 52, 2333 AK Leiden, The Netherlands; 7 Department of Child and Adolescent Psychiatry, GGZ Delfland, Center for Psychiatry, Amsterdam, The Netherlands; 8 Practice for Individual, Couple, and Family Therapy and Center for Training, De Kontekst, Van Breestraat 147HS, 1071 ZL Amsterdam, The Netherlands; 9 Research priority Area Yield, University of Amsterdam, Amsterdam, The Netherlands

**Keywords:** ADHD, Executive functioning, Depletion, Time-on-task, Reinforcement, Inhibition

## Abstract

Children with attention-deficit/hyperactivity disorder (ADHD) are characterized by deficits in their executive functioning and motivation. In addition, these children are characterized by a decline in performance as time-on-task increases (i.e., time-on-task effects). However, it is unknown whether these time-on-task effects should be attributed to deficits in executive functioning or to deficits in motivation. Some studies in typically developing (TD) adults indicated that time-on-task effects should be interpreted as depletion of executive resources, but other studies suggested that they represent depletion of motivation. We, therefore, investigated, in children with and without ADHD, whether there were time-on-task effects on executive functions, such as inhibition and (in)attention, and whether these were best explained by depletion of executive resources or depletion of motivation. The stop-signal task (SST), which generates both indices of inhibition (stop-signal reaction time) and attention (reaction time variability and errors), was administered in 96 children (42 ADHD, 54 TD controls; aged 9–13). To differentiate between depletion of resources and depletion of motivation, the SST was administered twice. Half of the participants was reinforced during second task performance, potentially counteracting depletion of motivation. Multilevel analyses indicated that children with ADHD were more affected by time-on-task than controls on two measures of inattention, but not on inhibition. In the ADHD group, reinforcement only improved performance on one index of attention (i.e., reaction time variability). The current findings suggest that time-on-task effects in children with ADHD occur specifically in the attentional domain, and seem to originate in both depletion of executive resources and depletion of motivation. Clinical implications for diagnostics, psycho-education, and intervention are discussed.

**Electronic supplementary material:**

The online version of this article (doi:10.1007/s00787-017-1006-y) contains supplementary material, which is available to authorized users.

## Introduction

Children with attention-deficit/hyperactivity disorder (ADHD) are characterized by inattention, hyperactivity, and/or impulsivity, which lead to problems in multiple domains. For example, children with ADHD have more academic problems [1] and adverse health outcomes [2], report lower quality of life [3], and usually have one or more comorbid psychiatric diagnoses [4]. Several models explaining ADHD have been proposed (see [5, 6]). One influential model is the dual pathway model, in which ADHD is characterized by deficits in both executive and motivational systems [7].

With regard to the executive pathway, several meta-analyses indicate that children with ADHD are impaired on multiple executive functions (EF) [8–11]. For example, response inhibition, which is regarded as one of the core, higher order executive functions [12, 13], has repeatedly shown to be implicated with ADHD [11, 14]. On a more basic pre-executive level, attention is a crucial prerequisite of executive functioning [13], and associations between ADHD and attentional problems are consistently reported (ranging from problems in sustaining attention on lab tasks to real life attention problems [9, 14]).

With respect to the motivational pathway, many empirical studies as well as theoretical models suggest aberrant motivation in children with ADHD (see [15] for an overview). Some models propose that children with ADHD have a higher reward sensitivity than controls (i.e., larger improvement in performance related to reward; [16, 17]), but experimental findings for this account are mixed [18]. However, a recent meta-analysis on reinforcement effects on inhibition in ADHD indicated that (1) a large majority of children, both with and without ADHD, benefited from reinforcement and (2) this reinforcement effect was stronger for ADHD (large effect size) than for controls (medium effect size), suggesting differential reward sensitivity between groups [16]. The authors note that only 24% of the studies found significant group × reinforcement interactions in this direction, which is in line with the mixed findings that were mentioned previously.

EF performance in children with ADHD is often more characterized by a stronger decrease in performance over time (time-on-task) as compared to TD controls [19]. It has been argued that these time-on-task effects originate in difficulties sustaining attention, which is a typical, although not specific [20], feature of ADHD [14, 21]. In accordance with the dual pathway model, this time-on-task effect can be caused by degraded (EF) resources, but it may also be possible that decreased levels of motivation explain this decrease in performance. It was shown that time-on-task effects on working memory in ADHD could be partly counteracted with reinforcement [22], suggesting that they should at least partly be attributed to decreased motivation. However, to our knowledge, it has never been tested before whether this is also the case for response inhibition and attention. Therefore, the current study investigates whether time-on-task effects on inhibition and attention in children with ADHD can be remedied by increasing motivation.

Dual pathway models of ADHD do not directly speak to the role of motivation on time-on-task effects. However, the effect of motivation on time-on-task effects is central in the literature on resource depletion in healthy adults. Some resource depletion theorists argue that self-control capacities, a concept highly related to EF [23], are limited, and consequently, self-control performance degrades after successive attempts (for reviews, see [24, 25]). However, others have argued that a decline in motivational resources (i.e., “reduced motivation to attain task goals” [26]) can also explain time-on-task effects [27, 28], as these effects appear to be weaker if participants are motivated [26, 29].

To sum up, the current study combines dual pathway models of ADHD and resource depletion models of time-on-task effects in healthy adults, to assess the origin of time-on-task effects in children with ADHD. That is, we test whether children with ADHD are more affected by time-on-task effects than TD children. To investigate the nature of these time-on-task effects, depletion of resources and depletion of motivation were disentangled. Children with and without ADHD performed twice on the stop-signal task (SST; [30, 31]), which yields a measure of response inhibition and more indirect measures of (in)attention. In the second task, participants were either assigned to a reinforced or a non-reinforced condition.

First, we hypothesize degraded performance of children with ADHD as compared to TD children in the first task and in the second task without reinforcement (effects of group) [8–11]. Second, we hypothesize degraded performance on the second task without reinforcement as compared to the first task (effect of time-on-task), and we expect this effect to be larger in children with ADHD than in TD controls (time × group interaction; [19]). Third, we hypothesize a better performance on the second task with reinforcement as compared to the second task without reinforcement (effect of reinforcement), and we hypothesize children with ADHD profit more from reinforcement than TD controls (reinforcement × group interaction; [16, 22]).

## Method

### Participants

ADHD participants were recruited from an academic outpatient mental healthcare center and TD control participants were recruited from elementary schools. In the ADHD group, children were included when they were diagnosed with ADHD (all subtypes), according to the assessment by expert psychologists or psychiatrists from the academic outpatient mental healthcare center, following DSM-IV-TR criteria [32]. There was no exclusion based on other disorders. Children in the control group were included only when their primary caretakers confirmed that there was no ADHD diagnosis. In total, our sample consisted of 111 children aged between 9 and 13 years. 54 children with ADHD (45 boys, mean age 11.2 years, SD = 1.04) and 57 children without ADHD (27 boys, mean age 11.8 years, SD = 0.68) were included.

When using stimulant medication, participants were instructed not to take their medication on the day of testing, to reach total washout [33]. Informed consent was obtained from primary caretakers of all children. All procedures were in accordance with the ethical standards of the institutional research committee and with the 1964 Helsinki declaration and its later amendments.

## Materials

Response inhibition and, indirectly, attention were measured with the standard stop-signal task (SST; [30, 31]), which is a reliable indicator of inhibition in children with ADHD [34]. Several studies showed associations between the SST and a wide range of real life behaviors, e.g., associations with classroom observations of children with ADHD [35], with teacher ratings of inattention [36], with observations as well as classroom measures of hyperactivity and inattention [37], and with inattention measured by both parents and teachers [38].

The SST was administered twice (i.e., T1 and T2), both administrations consisted of one practice block and four experimental blocks of 56 trials each. In this task, children have to press one out of two marked buttons on the keyboard, corresponding to green go signals appearing in the center of the screen, as fast and accurate as possible (i.e., the go task). In T1 [X] required a left response and [O] required a right response; these were replaced by [H] and [S], respectively, at T2 to prevent learning effects. A choice between two response keys is necessary to create time to process a potential stop signal. This presentation order was counterbalanced across participants (the first, third, fifth, etc. participant was assigned to [XO] at T1 and [HS] at T2, and in the second, fourth etc. participant, this was reversed). In 25% of the cases, the green go signal turned red, indicating that the response tendency had to be inhibited. To ensure that participants succeeded to inhibit their response in 50% of the cases, the time between the go signal and the stop signal was adaptive. That is, if stopping was successful, the interval between the go signal and the stop signal (i.e., the stop-signal delay) of the following stop trial was increased by 50 ms, making it harder to inhibit. On the other hand, if the participant failed to stop, the stop-signal delay of the following stop trial was shortened by 50 ms, making inhibition easier. Accordingly, the stop-signal reaction time (SSRT) can be estimated. This reflects the estimated mean time required to inhibit responses to stop signals, implying that a short SSRT indicates good inhibitory capacities. SSRT was calculated according to the integration method and the race model [41]. Assuming independence between go and stop processes, the finishing time of the stop process bisects the go RT distribution. Given that the response could not be stopped successfully on nth percent of all stop trials, SSRT is calculated by subtracting the mean stop-signal delay from the go RT that represents the nth percentile of go RTs (i.e., the finishing time of the stop process).

Furthermore, the SST provides an index of choice errors (i.e., pressing the wrong button), omission errors on go trials (i.e., no response within the response frame), and reaction time (including its variance). More omission errors, slower mean RT, and higher RT variability (defined as the standard deviation of all RTs of a participant within the SST) are associated with problems in the domain of attention [39]. More specifically, omission errors on a Go/NoGo task are related to symptoms of inattention in ADHD as reported by both caregivers and teachers [40]. Moreover, ADHD is characterized by attentional lapses, which generate reaction time distributions with a positive skew, leading to increased mean reaction times and higher RT variability [41, 42]. Relatedly, attention lapses appear to be related to errors and variability of reaction times, which is referred to as state instability [43]. Therefore, omission errors, RT and RT variability were taken as indirect measures of the basic attentional processes.

### Procedure

To induce time-on-task effects, the SST was administered twice in succession. Before every block, children were instructed to respond as fast and accurate as possible to go trials and to withhold their response if the signal turned red (i.e., not to press a button). To avoid adoptation of a waiting strategy, participants were also told that it was not allowed to wait for the signal to become red. In the second task, participants were randomly assigned to either a reinforcing or a non-reinforcing condition. In the reinforcing condition, children were told that they could earn coins which could be used to “buy” a present at the end of the task if they had earned at least ten coins. Reinforcement was not aimed at any specific aspect of the SST, and children were instructed to respond as fast and accurate as possible. Although the present was emotionally appealing for the participants, the monetary value was about 0.50 euro. To motivate participants, the box with presents was already shown before the beginning of the second task. After each block, children were informed on the screen about the amount of earned coins. Feedback on the amount of earned coins was manipulated; the cumulative amount of coins shown after each block was, respectively, 2, 5, 7, and 9 or 10.^1^ There was no possibility of losing in both conditions. The duration of one administration of the SST was approximately 16 min; the duration of the entire session ranged from 45 to 60 min. The time between the end of the first and the beginning of the second administration of the SST was approximately 2 min.

### Data analysis

A repeated measures design with one within-subjects factor (time, T1 and T2) and two between-subjects factors (ADHD vs. TD controls; reinforcement vs. no reinforcement at T2) was used. Note, however, that the design is not fully crossed, as none of the participants was reinforced during the first task. A fully crossed design would have led to power difficulties, given the limited availability of participants. Therefore, the current data could not be analyzed with a regular repeated measures analysis, but were analyzed with a multilevel analysis with time as a first level variable and group and reinforcement as second level variables [44]. Age was added as covariate, as it might be related to executive functioning [45]. Gender and intelligence were not added as covariates, because a higher proportion of boys and a lower average intelligence level are typical features of ADHD as compared to controls [46], and are, therefore, inherently not suitable as covariates [47] (see Appendix 1 for the complete multilevel model).

Five dependent variables were derived from the SST (see “Materials” section). Note that for the omission and choice errors, the square root of the raw scores was analyzed, because percentages generally are not normally distributed.

## Results

### Exclusion of participants

15 participants were excluded from the analyses (12 boys, 3 girls; mean age 11.2 years, SD = 1.18). Four of them were excluded due to procedural errors. In addition, 11 participants were excluded because of aberrant performance: one ADHD participant refused to continue with the second task; one ADHD participant made choice errors on more than 50% of the go trials (at T1), indicating difficulty in understanding the task; one ADHD participant had a very abnormal response pattern in which RT variability was larger than mean RT (at T2 without reinforcement); four participants were excluded, because SSRT was below 100 ms, indicating that SSRT estimation was not reliable for these participants (one ADHD participant at T1; two ADHD participants at T2, without reinforcement; and one TD participant at T2 with reinforcement); four participants were excluded, because the standardized value of at least one of the outcome measures was more than three standard deviations from the average (one ADHD participant had abnormal slow reaction times at T1 as well as T2 without reinforcement;^2^ one ADHD participant had an abnormal high SSRT at T1;^3^ one ADHD participant had an abnormal high SSRT at T2,^4^ without reinforcement; and one TD participant had an abnormal high SSRT at T2,^5^ without reinforcement).

Interestingly, 9 of the 11 participants excluded because of aberrant performance were diagnosed with ADHD. Most of these participants were excluded because of aberrant performance in the second task without reinforcement: of the seven exclusions based on performance at T2, two were reinforced, and five were not.

### Demographics

After exclusion, the sample consisted of 96 participants (42 ADHD, 54 TD children). Differences between the ADHD group and the TD control group were significant with regard to age. The mean age was 11.2 (SD = 1.0) and 11.8 (SD = 0.68) years for ADHD and TD controls, respectively: *t* (94) = 3.21, *p* = 0.002). Therefore, age was added as covariate in all further analyses. Groups also differed in gender (83% vs. 46% boys in ADHD and TD, respectively: *χ* ^*2*^ (1) = 13.8, *p* < 0.001). For extra analyses with gender added as covariate, see Appendix 2.^6^

In the ADHD group, 31 children were diagnosed with the combined type, 5 with the inattentive type, 2 with the hyperactive-impulsive type, and 4 children were diagnosed with ADHD not otherwise specified. With regard to comorbidity, 24 children had no comorbid diagnosis, 7 children had a comorbid learning disorder, 5 had a comorbid disruptive behavior disorder (i.e., conduct disorder, oppositional defiant disorder, or disruptive behavior disorder not otherwise specified), 3 had a comorbid parent–child relational problem, 2 had a comorbid mood disorder, and 1 had a comorbid communication disorder.

### Effects of group: ADHD vs. TD controls (T1)

To test our first hypothesis that EF on T1 is impaired in children with ADHD as compared to TD controls, we performed ANCOVAs (and thus not a multilevel analysis) for all outcome measures in which we controlled for age differences between groups. The assumption of parallel regression lines was not violated for all five outcome measures (*p* > 0.05). ANCOVAs revealed only group differences at baseline for reaction time variability [*F* (1, 93) = 10.8, *p* = 0.001] and omission errors [*F* (1, 93) = 4.5, *p* = 0.04]. No differences were found for SSRT [*F* (1, 93) = 2.7, *p* = 0.11], mean reaction time [*F* (1, 93) = 0.001, *p* = 0.98], and choice errors [*F* (1, 93) = 2.9, *p* = 0.10] (see Table 1 for means and standard deviations at T1).

**Table 1:** Group means at T1 and at T2, with and without reinforcement

Measure	TD (T1)	TD (T2) −	TD (T2) +	ADHD (T1)	ADHD (T2) −	ADHD (T2) +
Number of participants	54	28	26	42	16	26
Reaction time in ms (SD)	730 (175)	670 (130)	712 (228)	730 (161)	741 (98)	767 (206)
Reaction time variability in ms (SD)	203 (52)	192 (44)	177 (49)	237 (48)	254 (45)	230 (58)
% choice errors (SD)	3.49 (3.91)	3.38 (3.46)	3.27 (3.18)	5.90 (5.34)	4.76 (3.76)	5.24 (4.64)
% omission errors (SD)	2.38 (3.13)	0.98 (1.34)	2.40 (3.74)	3.83 (3.74)	5.84 (5.73)	5.91 (6.17)
SSRT in ms (SD)	254 (65)	260 (53)	246 (62)	282 (75)	269 (54)	243 (54)

Symbols + and – represent conditions with and without reinforcement

Note that this table depicts the  % of choice and omission errors, whereas the square root is used in the analyses. Note that the number of ADHD participants in the conditions without and with reinforcement is unequal, as a relatively high number of participants that did not receive feedback were excluded because of aberrant performance. Reaction time variability reflects the mean within subject variability in RTs, whereas the SD of reaction time reflects the standard deviation of the mean RTs of all subjects

*TD* typically developing control group, *ADHD* attention-deficit/hyperactivity disorder, *ms* milliseconds, *SSRT* stop-signal reaction time, *SD* standard deviation

After the baseline assessment at T1, participants were randomly assigned to the second task with or without reinforcement. At baseline, there were no significant differences on all five outcome measures between children assigned to the reinforced and non-reinforced second task. This was tested by adding the reinforcement condition as an additional between factor to the ANCOVAs.

### Multilevel analyses (also see Table 2)

#### Effects of group: ADHD vs. TD controls (T2: not reinforced)

When comparing ADHD and TD control groups at T2 (without reinforcement), the multilevel analysis indicated that participants with ADHD had a larger reaction time variability [*t* (119.62) = −5.04, *p* < 0.001] and made more omission errors [*t* (124.88) = −4.70, *p* < 0.001]. No differences between ADHD and TD controls at T2 were found for SSRT, mean reaction time, and choice errors.

**Table 2:** Overview of all effects in the multilevel model

γ	γ_01_	γ_10_	γ_11_	γ_02_	γ_03_	γ_04_
Variable	Group	Time	Group × time	Reinforcement	Group * reinforcement	Age
SSRT	*B* = 8.2 (16.2), *p* = 0.61	*B* = 23.1 (12.6), *p* = 0.07	*B* = −30.7 (16.1), *p* = 0.06	*B* = −9.8 (14.3), *p* = 0.50	*B* = −7.4 (18.9), *p* = 0.70	*B* = −0.90 (0.55), *p* = 0.11
Mean RT	*B* = −58.0 (49.1), *p* = 0.24	*B* = 7.8 (32.5), *p* = 0.81	*B* = 58.6 (40.8), *p* = 0.15	*B* = 56.8 (40.4), *p* = 0.16	*B* = −2.4 (53.2), *p* = 0.96	*B* = 0.008 (1.66), *p* = 0.996
RT Var.	*B* = −71.7, (14.2), *p* < 0.001***	*B* = −23.0 (10.2), *p* = 0.03*	*B* = 36.1 (12.9), *p* < 0.01**	*B* = −33.9 (12.4), *p* < 0.01**	*B* = 24.1 (16.4), *p* = 0.14	*B* = 0.13 (0.47), *p* = 0.79
Omission errors	*B* = −1.40 (0.30), *p* < 0.001***	*B* = −0.54 (0.20), *p* = 0.01*	*B* = 0.95 (0.26), *p* < 0.001***	*B* = −0.18 (0.25), *p* = 0.48	*B* = 0.38 (0.33), *p* = 0.25	*B* = 0.004 (0.01), *p* = 0.72
Choice errors	*B* = −0.18 (0.27), *p* = 0.50	*B* = 0.11 (0.20), *p* = 0.58	*B* = −0.29 (0.26), *p* = 0.27	*B* = 0.07 (0.24), *p* = 0.78	*B* = −0.34 (0.31), *p* = 0.28	*B* = −0.001 (0.01), *p* = 0.23

*SSRT* stop-signal reaction time, *RT* reaction time, *Var*. variability. B (SE) represents the unstandardized estimate with its standard error, *γ*_*01*_ the group effect at T2 without reinforcement, *γ*_*10*_ the time effect in ADHD without reinforcement, *γ*_*11*_ the interaction effect between group and time without reinforcement, *γ*_*02*_ the reinforcement effect at T2 in ADHD, *γ*_*03*_ the interaction effect of group and reinforcement at T2, *γ*_*04*_ represents the effect of age in boys with ADHD at T2 receiving no reinforcement

* *p* < 0.05, ** *p* < 0.01, *** *p* < 0.001

#### Time-on-task effects in ADHD (T2 vs. T1: not reinforced)

Children with ADHD were characterized by increased reaction time variability [*t* (102.07) = −2.25, *p* = 0.027] and more omission errors [*t* (96.86) = −2.64, *p* = 0.010] on the second task without reinforcement, as compared to the first task. No differences between T2 and T1 in ADHD were found for SSRT mean reaction time and choice errors. Time-on-task effects were thus only found on indices of attention, but not on inhibition.

#### Time-on-task effects in ADHD vs. TD controls (T2 vs. T1: not reinforced)

**Fig. 1:**
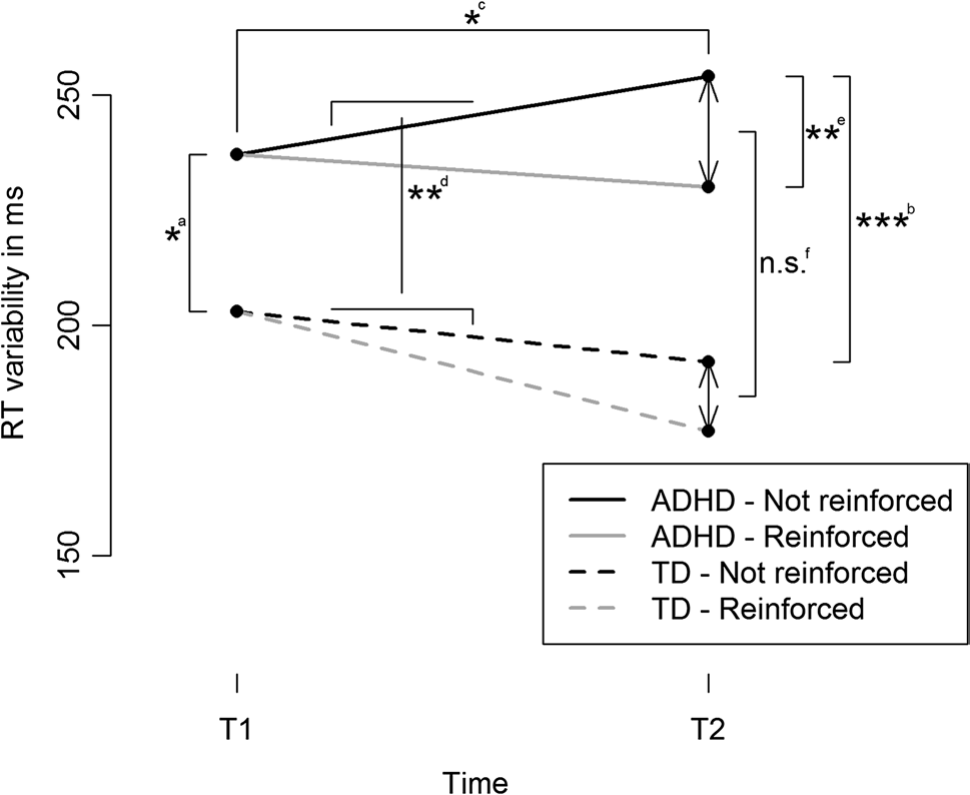
Reaction time variability as a function of time, group, and reinforcement condition. **a** effect of group at baseline (T1); **b** effect of group at T2 (non-reinforced); **c** effect of time in ADHD (non-reinforced); **d** time × group interaction (non-reinforced); **e** effect of reinforcement in ADHD; **f** group × reinforcement interaction. *p < 0.05, **p < 0.01, ***p < 0.001. *ADHD* attention-deficit/hyperactivity disorder, *TD* typically developing control group, *ms* milliseconds, *n.s.* not significant, *RT* reaction time

A significant time × group interaction was observed for reaction time variability [*t* (102.28) = 2.80, *p* = 0.006]. As shown above, in the ADHD group, reaction time variability increased on the second as compared to the first task. The positive *t* value for the interaction effect indicates that this effect was less pronounced in the TD group. Visual inspection of the effect (Fig. 1) suggests that in the TD group, reaction time variability even decreased in the second as compared to the first task. Similarly, a significant time × group interaction was observed for omission errors [*t* (97.02) = 3.70, *p* < 0.001]. In children with ADHD, the amount of omission errors increased on the second as compared to the first task (see analyses above), whereas visual inspection suggests that the amount of omission errors in the TD control group even decreased (Fig. 1). No time × group interactions were found for SSRT, mean reaction time, and choice errors. To sum up, this indicates larger time-on-task effects in the ADHD group than in the TD control group on indices of attention, but not inhibition.

#### Effects of reinforcement in ADHD (T2: reinforced vs. not reinforced)

Children with ADHD who were vs. were not reinforced were characterized by lower RT variability [*t* (91.95) = −2.73, *p* = 0.008]. Reinforcement did not influence SSRT, mean reaction time, omission errors, and choice errors. This indicates that children with ADHD performed better when they were reinforced as compared to when they were not reinforced, but only with regard to RT variability.

#### Effects of reinforcement in ADHD vs. TD controls (T2: reinforced vs. not reinforced)

No significant group × reinforcement effect was found for all measures, indicating that reinforcement equally influenced children with and without ADHD.

## Discussion

Dual pathway models of ADHD and depletion theories were combined to investigate potential depletion of executive resources and depletion of motivation in children with and without ADHD. The SST was administered twice in school-aged children with and without ADHD, in which half of the participants was reinforced during the second task. We hypothesized (1) degraded performance of children with ADHD as compared to typically developing (TD) children on the first task and the second task without reinforcement. Moreover, we expected (2a) degraded performance on the second task without reinforcement, as compared to the first task and (2b), and we expected this effect to be larger in children with ADHD than in TD children. We expected (3a) improved performance on the second task with reinforcement, as compared to the second task without reinforcement and (3b), and we expected that children with ADHD profited more from reinforcement than TD children. Performance was measured at two levels, with response inhibition as core higher order executive function, and at a more basic pre-executive level [13], attentional indices as reaction time (RT), RT variability, and errors.

With respect to the first hypothesis, at baseline, groups differed with regard to reaction time (RT) variability and omission errors: children with ADHD were characterized by larger RT variability and made more omission errors than children without ADHD. On the second task without reinforcement, groups differed on RT variability and omission errors: children with ADHD had larger RT variability and made more omission errors than children without ADHD.

No difference between groups was found for SSRT. Although the typical finding of inhibitory differences between children with and without ADHD is quite robust [9, 16, 48, 49], there are other studies that did not find this difference either [50–53].

Potential explanations might relate to participant characteristics. For example, only a subgroup of children with ADHD is characterized by inhibitory deficits [54–56]. Furthermore, comorbidity profiles seem to play a role. For instance, no inhibitory differences were found between control children and children with only ADHD (i.e., without comorbid disorders; [52]). In our sample, comorbidity occurred less frequently than generally described in ADHD: 57% of our ADHD group had no comorbid disorder, whereas other literature indicates that approximately two-third of the children with ADHD have at least one other comorbid disorder [4]. In line with this explanation, the average SSRT at baseline in our sample was 254 ms for controls, 274 ms for children with ADHD without comorbid disorders, and 293 ms for children with ADHD comorbid disorder(s). An additional analysis proved that children with ADHD without comorbid disorder(s) did not differ from controls with regard to SSRT at T1 [*F* (1,76) = 1.4, *p* = 0.24], whereas children with ADHD and comorbid disorder(s) had higher SSRTs than controls [*F* (1,70) = 4.7, *p* = 0.03].^7^

Relatedly, Daugherty and colleagues [51] explain differences between studies in terms of severity of the clinical group, where more severe ADHD groups are most likely to show pronounced inhibitory dysfunctioning. In sum, lower comorbidity rates as well as lower severity of ADHD symptoms (e.g., we included four children with ADHD not otherwise specified) might be an explanation for the absence of inhibition effects between groups.

However, increased RT variability in children with ADHD as compared to TD control children was apparent both at baseline and on the second administration without reinforcement. This concurs with a large body of literature, arguing that increased RT variability is a typical and robust finding in ADHD (see [57] for an excellent extensive meta-analytic review). Some theorists even argue that RT variability is a causal mechanism in the existence of ADHD (e.g., Default Mode Network Model [21]), while others regard this variability as the result of other underlying mechanisms, such as behavioral inhibition [14, 57].

With respect to the second hypothesis, the expected time-on-task effect in ADHD was partially confirmed: RT variability and omission errors increased on the second task as compared to the first in the ADHD group. In line with our hypothesis, this time-on-task effect on RT variability and omission errors was larger in the ADHD than in the TD control group (in fact, the TD control group did not deteriorate at all). RT variability and omission errors have been linked to problems with sustained attention and attentional lapses [39, 40, 42, 58]. All together, these results indicate that time-on-task effects in ADHD mainly seem to occur within the domain of basic attention, and not on response inhibition.

With respect to the third hypothesis, the effect of reinforcement in ADHD was partly as expected: reinforced children with ADHD had a smaller RT variability as compared to children with ADHD who were not reinforced in the second task, which might indicate that reinforcement prevented attentional lapses. This finding is in line with a recent meta-analytic review that found small improvements in RT variability as a result of external reinforcement [57].

However, no effect of reinforcement was found on all other outcome indices. This implies that, among all outcome variables, RT variability might be particularly sensitive to the effects of reinforcement. Furthermore, discordant to expectations, reinforcement effects did not differ between groups for all indices, implying that children with ADHD did not profit more from reinforcement than their typically developing peers. This contradicts several studies, reporting that children with ADHD profited more from reinforcement (see [16] for a meta-analysis), but concurs with others that also did not find such differences between ADHD and typically developing controls [53, 59–63]. Moreover, despite the overall significant effect, the same meta-analysis showed that only 24% of the studies found a larger reward sensitivity in ADHD as compared to controls [16].

One explanation for the limited effects of reinforcement could be that reinforcement in the current study was not strong enough. Although the gift was emotionally appealing, it was only worth 50 eurocents. Children with ADHD are assumed to have an elevated reward threshold [18, 64], and as shown by Dovis and colleagues [22], only relatively large rewards (above threshold) motivated children with ADHD enough to improve their performance on EF tasks, whereas small rewards did not exert any influence on performance. A second explanation for the absence of pronounced motivation effects is that several studies have pointed out that only a minority of children with ADHD shows abnormal sensitivity to reinforcement [65, 66], possibly explaining the limited reinforcement effects at group level in the current study.

To summarize, stronger time-on-task effects were found in children with ADHD as compared to children without ADHD on indices of basic attention (i.e., RT variability and omission errors). In terms of depletion theories, reinforcement prevented a time-on-task effect on RT variability, implying that the time-on-task effect in the non-reinforced condition could be, at least partly, explained by a depletion of motivation. On the other hand, reinforcement did not affect the time-on-task effect on omission errors, implying that this time-on-task effect could be driven by a depletion of executive resources. Finally, no time-on-task effects were found on other indices, among which inhibition. This implies that, compared to lower level attentional capacities, higher order executive functions, such as inhibition, seem to be less susceptible to the effects of depletion of executive resources and depletion of motivation.

In the current study, slower mean RTs and higher RT variability (i.e., derived from a Gaussian model with two parameters: mean and variance) were interpreted as potential indicators of attention lapses. However, as attention lapses produce a skew in RT distributions, an ex-Gaussian model might be more appropriate, in which a third parameter (τ) indexes this skew specifically [67] (note, however, that the τ parameter could be interpreted as an indicator for many different cognitive processes [68]). Therefore, all multilevel analyses were also performed for τ as an index for attention lapses.^8^ The only significant finding was related to reinforcement: more attentional lapses were reported when participants were not reinforced instead of reinforced at T2 [*t* (91.24) = −2.02, *p* < 0.05]. Therefore, this additional analysis partly supports our conclusion that problems (in this case attention lapses) originating in a depletion of motivation can be counteracted with reinforcement.

The current results should be considered in the light of four limitations. First, 15 children were excluded, 11 of them because of aberrant performance. Nine of those 11 were diagnosed with ADHD, and a majority dropped out on the second task (mostly without reinforcement). Conceivably, these excluded participants had most pronounced symptoms, and effects were larger if data from these children were taken into account (see Appendix 3 for results without excluding any of the participants). These testing difficulties demonstrate an obvious limitation of the depletion paradigm, in which multiple monotonous tasks are administered in succession, in samples with ADHD. This is in line with a recent meta-analytic review, which described an increase in core ADHD symptomatology (i.e., hyperactivity) in highly cognitive demanding situations as compared to situations with low cognitive demands [69]. Therefore, on the other hand, the current depletion paradigm might be an promising way of testing, mirroring the daily life routine at school, and creating a situation in which ADHD symptoms come to light easier.

Second, it would have been more optimal to diagnose ADHD participants by means of a semi-structured clinical interview, as the currently used clinical diagnoses might be more lenient and potentially included some children with ‘subclinical’ ADHD. Relatedly, clinical diagnoses might be subject to biases [70]. However, it should be noted that stricter diagnostics and exclusion of subclinical ADHD participants would logically result in larger and not smaller differences between typically developing controls and ADHD subjects. This limitation could also apply to the control group, in which potential neuropsychiatric problems could have been missed. However, the average SSRT of the control group (at T1, SSRT = 254 ms) was consistent with typical control group SSRTs reported in a meta-analysis ([11]; the mean SSRT in control groups on similar SSTs was 284 ms (*k* = 24, *n* = 937). Hence, inhibitory problems in the control group seem unlikely.

Third, the absence of reinforcement effects is interpreted as evidence in favor of executive over motivational depletion. However, self-evidently, another explanation for the absence of those effects could be that statistical power was not high enough. Therefore, these results should be interpreted with caution.

Fourth, the validity of the SSRT as a pure index of response inhibition is subject to debate. This SSRT deficit might reflect general attentional or cognitive processes that go beyond purely inhibitory processes [8, 49]. However, on a more theoretical level, the current SST is, in our opinion, the best index to assess response inhibition. First, it directly taps into the construct of interest and measures inhibition over a longer time period. Second, a meta-analysis on response inhibition differences between ADHD and controls reviewing 41 studies showed that between group differences (i.e., ADHD groups showing stronger inhibitory deficits) were most pronounced when responses, as in the current study, were spatially noncompatible [11]. Although note that some of the studies reported in that meta-analysis also reported no group differences in SSRT, as in the current study. Third, an additional advantage of the SST is that it derives both measures for inhibition and for attention.

The current study has several implications, for future research as well as for clinical practice. EF was assessed under depleting circumstances, which matches most children’s daily school routine, in which they have to use their EFs over prolonged periods of time. This is not to say that we advocate the SST as an ecologically valid EF instrument, as laboratory EF tasks do not correlate well with real life measures [71] and the task that was used in the current study was more repetitive than regular schoolwork.

Our results showed that attentional problems in ADHD became more apparent in the second task. Moreover, the relatively large drop-out in our study potentially indicates that core psychiatric symptoms might come to light easier after a certain amount of time. Hence, we recommend future research to further investigate the diagnostic value of this paradigm in clinical samples.

With regard to treatment of ADHD, psycho-education to children with ADHD and their parents and teachers should emphasize the tendency for problems to increase after prolonged exertion. If children recognize depletion, they can be taught to adjust their behavior accordingly, for example, by taking a short break or switch to less demanding tasks. Furthermore, we suggest therapists to keep their sessions short and offer short breaks, to prevent attentional lapses. If time-on-task effects occur, for example, during school work, this can be partly counteracted with reinforcement.

The current approach was adopted to distinguish between depletion of executive resources and depletion of motivation. However, recent studies show that children with ADHD show heterogeneous patterns of deficits, some having especially deficits in executive functioning, whereas in others, mainly, motivational aberrations are observed [56, 65, 66, 72, 73]. Consequently, the use of a design comparing ADHD and control groups limits the possibility to elucidate this heterogeneity within the ADHD group. A more personalized approach, in which individual deficits could be assessed and, eventually, treated, would be a promising addition to the literature and is in line with current trends in youth mental healthcare ([73]; see [74] for an extensive review on personalized interventions for youth).

In sum, children with ADHD are more affected time-on-task effects than TD controls, as shown on measures of inattention (RT variability and omission errors), but not inhibition. These time-on-task effects seem to originate in a depletion of executive resources as well as a depletion of motivation. Offering external reinforcement is a promising way to compensate for depletion of motivation and, consequently, to prevent attention lapses in children with ADHD. The depletion paradigm offers both a new perspective on diagnostic assessment of ADHD and provides further clues for optimizing treatment of children with ADHD.

## Electronic supplementary material

Below is the link to the electronic supplementary material.

Supplementary material 1 (DOCX 19 kb)

Supplementary material 2 (DOCX 22 kb)

Supplementary material 3 (DOCX 21 kb)

## Footnotes

1 The end score was manipulated to either 9 or 10. This in order to prevent the spread of the rumor that all children had won a present regardless of their performance, as TD controls usually had class together. After all data were gathered, children that ended up with 9 points received a present as well.

2 Mean reaction times of this participant were 1288 and 1219 ms at T1 and T2, respectively.

3 SSRT at T1 of this participant was 678 ms.

4 SSRT at T2 of this participant was 677 ms.

5 SSRT at T2 of this participant was 538 ms.

6 Note however that the addition of gender as covariate yielded no differences in the results of the multilevel analyses (see extra analyses in Appendix 2).

7 Note however that children with ADHD with and without comorbidity did not differ significantly on SSRT at baseline.

8 These analyses were performed using the retimes package in R.
