# Supplementary material for: Time-on-task effects in children with and without ADHD: depletion of executive resources or depletion of motivation?
Source: Eur Child Adolesc Psychiatry. 2017 May 23;26(12):1471–81. doi: 10.1007/s00787-017-1006-y (PMC5701950; doi:10.1007/s00787-017-1006-y)
Supplement: Supplementary file 1 — Supplementary material 1 (DOCX 19 kb) [file 787_2017_1006_MOESM1_ESM.docx]

**Appendix 1 – multilevel model**

The multilevel model, with Y denoting the outcome for time *i* and participant *j*, is as follows:

*Level 1:* Y_ij_ = β_0j_ + β_1j_ * time_ij_ + ε_ij_

*Level 2:* β_0j_ = γ_00_ + γ_01_ * group_j_ + γ_02_ * reinforcement_j_ + γ_03_ * group_j_ * reinforcement_j_ + γ_04_ * age_j_

β_1j_ = γ_10_ + γ_11_ * group_j_

*Combined:* Y_ij_ = γ_00_ + γ_01_ * group_j_ + γ_02_ * reinforcement_j_ + γ_03_ * group_j_ * reinforcement_j_ + γ_04_ * age_j_ + γ_10_ * time_ij_ + γ_11_ * group_j_ * time_ij_ + ε_ij_

Note that both the error (ε) variances at T1 and T2, as well as their covariance were estimated to account for the repeated measures nature of the data.

In order to obtain tests of our hypotheses, we code time as 0 for the second task (T2) and 1 for the first task (T1).^[[1]](#footnote-1)^ Group is coded 0 for ADHD and 1 for TD controls^[[2]](#footnote-2)^. Reinforcement is coded 0 for no reinforcement and 1 for reinforcement. Age was mean centered and coded continuously, implying that a value of 0 represents the mean age of the sample. Given this coding, the crucial parameters in the model have the following interpretation: γ_01_ represents the group effect at T2 without reinforcement, γ_02_ the reinforcement effect at T2 in ADHD, γ_03_ the interaction effect of group and reinforcement at T2, γ_10_ the time effect in ADHD without reinforcement, and γ_11_ the interaction effect between group and time without reinforcement.^[[3]](#footnote-3)^

1. Note that because of this coding, for time comparisons, negative t-values represent higher values at T2. [↑](#footnote-ref-1)
2. Note that because of this coding, for group comparisons, negative t-values represent higher values in the ADHD group. [↑](#footnote-ref-2)
3. Furthermore, γ_00_ represents the value for an averagely aged participant with ADHD at T2. γ_04_ represents the effect of age in participants with ADHD at T2 receiving no reinforcement. [↑](#footnote-ref-3)
