# Supplementary material for: Time-on-task effects in children with and without ADHD: depletion of executive resources or depletion of motivation?
Source: Eur Child Adolesc Psychiatry. 2017 May 23;26(12):1471–81. doi: 10.1007/s00787-017-1006-y (PMC5701950; doi:10.1007/s00787-017-1006-y)
Supplement: Supplementary file 2 — Supplementary material 2 (DOCX 22 kb) [file 787_2017_1006_MOESM2_ESM.docx]

**Appendix 2. Multilevel model with gender as additional covariate**

The multilevel model, with gender added as covariate, with Y denoting the outcome for time *i* and participant *j*, is as follows:

*Level 1:* Y_ij_ = β_0j_ + β_1j_ * time_ij_ + ε_ij_

*Level 2:* β_0j_ = γ_00_ + γ_01_ * group_j_ + γ_02_ * reinforcement_j_ + γ_03_ * group_j_ * reinforcement_j_ + γ_04_ * age_j_ + γ_05_ * gender_j_

β_1j_ = γ_10_ + γ_11_ * group_j_

*Combined:* Y_ij_ = γ_00_ + γ_01_ * group_j_ + γ_02_ * reinforcement_j_ + γ_03_ * group_j_ * reinforcement_j_ + γ_04_ * age_j_ + γ_05_ * gender_j_ + γ_10_ * time_ij_ + γ_11_ * group_j_ * time_ij_ + ε_ij_

Gender is coded 0 for boys and 1 for girls. The results of all multilevel analyses with gender as covariate are depicted in the table below. Note that there are no significant effects for the model with gender as covariate that were not significant in the model without gender as covariate and vice versa.

**Table A1.** Overview of all effects in the multilevel model (N=96)

| **γ** | **γ_01_** | **γ_10_** | **γ_11_** | **γ_02_** | **γ_03_** | **γ_04_** | **γ_05_** |
| --- | --- | --- | --- | --- | --- | --- | --- |
| Variable | Group | Time | Group * Time | Reinforcement | Group * Reinforcement | Age | Gender |
| SSRT | B = 11.5 (17.0),  *p* = .50 | B = 23.1 (12.7),  *p* = .07 | B = -30.7 (16.1),  *p* = .06 | B = -9.8 (14.4),  *p* = .50 | B = -7.4 (18.9),  *p* = .70 | B = -.95 (.56),  *p* = .09 | B = -8.0 (12.4), *p* = .52 |
| Mean RT | B = -55.1 (51.6),  *p* = .29 | B = 7.8 (32.5),  *p* = .81 | B = 58.6 (40.8),  *p* = .15 | B = 56.9 (40.4),  *p* = .16 | B = -2.5 (53.3),  *p* = .96 | B = -.04 (1.69),  *p* = .98 | B = -6.7 (37.3), *p* = .86 |
| RT Var. | B = -69.6, (14.9),  *p* < .001*** | B = -23.1 (10.3),  *p* = .03* | B = 36.2 (12.9),  *p* < .01** | B = -34.1 (12.5),  *p* < .01** | B = 24.4 (16.5),  *p* = .14 | B = .09 (.47),  *p* = .85 | B = -5.5 (10.4), *p* = .60 |
| Omission errors | B = -1.30 (.31),  *p* < .001*** | B = -.54 (.20),  *p* = .01* | B = .95 (.26),  *p* < .001*** | B = -.18 (.25),  *p* = .48 | B = .38 (.33),  *p* = .26 | B = .002 (.01),  *p* = .85 | B = -.25 (.22),  *p* = .26 |
| Choice errors | B = -.10 (.29),  *p* = .72 | B = .11 (.20),  *p* = .58 | B = -.29 (.26),  *p* = .27 | B = .06 (.24),  *p* = .79 | B = -.34 (.31),  *p* = .28 | B = -.01 (.01),  *p* = .19 | B = -.20 (.21),  *p* = .35 |

* *p* < .05, ** *p* < .01, *** *p* < .001, SSRT = Stop Signal Reaction Time, RT = reaction time, Var. = Variability. B (SE) represents the unstandardized estimate with its standard error.γ_01_ represents the group effect at T2 without reinforcement, γ_10_ the time effect in ADHD without reinforcement, γ_11_ the interaction effect between group and time without reinforcement, γ_02_ the reinforcement effect at T2 in ADHD, γ_03_ the interaction effect of group and reinforcement at T2, γ_04_ represents the effect of age in boys with ADHD at T2 receiving no reinforcement and γ_05_ represents the effect of gender in averagely aged children with ADHD at T2 receiving no reinforcement.
