# Supplementary material for: Time-on-task effects in children with and without ADHD: depletion of executive resources or depletion of motivation?
Source: Eur Child Adolesc Psychiatry. 2017 May 23;26(12):1471–81. doi: 10.1007/s00787-017-1006-y (PMC5701950; doi:10.1007/s00787-017-1006-y)
Supplement: Supplementary file 3 — Supplementary material 3 (DOCX 21 kb) [file 787_2017_1006_MOESM3_ESM.docx]

**Appendix 3. Multilevel analyses without exclusions**

In the table below, the results of the multilevel analyses without excluding the participants mentioned under “Exclusion of participants” in the main manuscript are depicted. As can be seen by comparing the table below with Table 2 in the main manuscript, effects were generally larger when these participants were not excluded.

**Table A2.** Overview of all effects in the multilevel model (N=110)^[[1]](#footnote-1)^

| **γ** | **γ_01_** | **γ_10_** | **γ_11_** | **γ_02_** | **γ_03_** | **γ_04_** |
| --- | --- | --- | --- | --- | --- | --- |
| Variable | Group | Time | Group * Time | Reinforcement | Group * Reinforcement | Age |
| SSRT | B = -35.1 (28.9), *p* = .23 | B = -13.5 (20.5), *p* = .51 | B = -2.97 (28.2), *p* = .92 | B = -55.8 (27.4), *p* = .04* | B = 17.8 (38.0),  *p* = .64 | B = -.68 (.74), *p* = .36 |
| Mean RT | B = -46.9 (44.4), *p* = .29 | B = 40.3 (26.4),  *p* = .13 | B = 22.0 (36.4),  *p* = .55 | B = 79.4 (35.7),  *p* = .03* | B = -20.3 (49.7), *p* = .68 | B = -1.1 (1.5), *p* = .46 |
| RT Var. | B = -87.0, (14.0), *p* < .001*** | B = -35.2 (8.8),  *p* < .001*** | B = 47.9 (12.2),  *p* < .001*** | B = -47.5 (12.0), *p* < .001*** | B = 44.1 (16.7),  *p* = .01* | B = -.05 (.44), *p* = .91 |
| Omission errors | B = -1.57 (.28),  *p* < .001*** | B = -.39 (.16),  *p* = .02* | B = .77 (.23),  *p* = .001** | B = -.21 (.22),  *p* = .34 | B = .42 (.30),  *p* = .17 | B = -.01 (.01), *p* = .37 |
| Choice errors | B = -.87 (.30),  *p* < .01** | B = -.40 (.22),  *p* = .07 | B = .27 (.31),  *p* = .39 | B = -.61 (.28),  *p* = .03* | B = .29 (.39),  *p* = .45 | B = -.01 (.01), *p* = .31 |

* *p* < .05, ** *p* < .01, *** *p* < .001, SSRT = Stop Signal Reaction Time, RT = reaction time, Var. = Variability. B (SE) represents the unstandardized estimate with its standard error.γ_01_ represents the group effect at T2 without reinforcement, γ_10_ the time effect in ADHD without reinforcement, γ_11_ the interaction effect between group and time without reinforcement, γ_02_ the reinforcement effect at T2 in ADHD, γ_03_ the interaction effect of group and reinforcement at T2, γ_04_ represents the effect of age in boys with ADHD at T2 receiving no reinforcement and γ_05_ represents the effect of gender in averagely aged children with ADHD at T2 receiving no reinforcement.

1. One of the excluded participants quit after the first assessment. Therefore, this participant could not be taken into account in these analyses. Hence, N=110 instead of N=111. [↑](#footnote-ref-1)
